# Role of endothelial cells in vascular calcification

**DOI:** 10.3389/fcvm.2022.895005

**Published:** 2022-07-19

**Authors:** Han Jiang, Lihua Li, Lili Zhang, Guangyao Zang, Zhen Sun, Zhongqun Wang

**Affiliations:** ^1^Department of Cardiology, Affiliated Hospital of Jiangsu University, Zhenjiang, China; ^2^Department of Pathology, Affiliated Hospital of Jiangsu University, Zhenjiang, China

**Keywords:** endothelial cell (EC), endothelial-to-mesenchymal transition (EndMT), vascular calcification (VC), atherosclerosis (AS), endothelia cell dysfunction

## Abstract

Vascular calcification (VC) is active and regulates extraosseous ossification progress, which is an independent predictor of cardiovascular disease (CVD) morbidity and mortality. Endothelial cells (ECs) line the innermost layer of blood vessels and directly respond to changes in flow shear stress and blood composition. Together with vascular smooth muscle cells, ECs maintain vascular homeostasis. Increased evidence shows that ECs have irreplaceable roles in VC due to their high plasticity. Endothelial progenitor cells, oxidative stress, inflammation, autocrine and paracrine functions, mechanotransduction, endothelial-to-mesenchymal transition (EndMT), and other factors prompt ECs to participate in VC. EndMT is a dedifferentiation process by which ECs lose their cell lineage and acquire other cell lineages; this progress coexists in both embryonic development and CVD. EndMT is regulated by several signaling molecules and transcription factors and ultimately mediates VC *via* osteogenic differentiation. The specific molecular mechanism of EndMT remains unclear. Can EndMT be reversed to treat VC? To address this and other questions, this study reviews the pathogenesis and research progress of VC, expounds the role of ECs in VC, and focuses on the regulatory factors underlying EndMT, with a view to providing new concepts for VC prevention and treatment.

## Introduction

Vascular calcification (VC) is an independent predictor of cardiovascular disease (CVD) morbidity and mortality. Its continuous development induces reduced blood vessel compliance, the rupture of atherosclerotic plaques, and thrombosis and poses obstacles to interventional surgery (1). Large-scale clinical epidemiological investigations have shown that VC gradually worsens with age; the incidence of VC in men > 70 years reaches 90% (2). Within the context of diabetes mellitus (DM), chronic kidney disease (CKD), and atherosclerosis (AS), VC incidences in young patients have also increased dramatically (1, 3). VC plays a key role in the progression of vascular injury and thus is an important direction for drug therapy; however, no specific therapeutic methods exist for VC. For a considerable period, research on VC has primarily focused on vascular smooth muscle cells (VSMCs), but recent studies have indicated that endothelial cells (ECs) are also involved in VC regulation and formation (4–6).

The vascular endothelium originates from the mesoderm. Physiologically, ECs maintain the integrity of the endothelium and ensure vascular homeostasis by adjusting metabolism and platelet activity (7). Due to their unique location, ECs first respond to flow shear stress (FSS) and various signaling molecules in the blood; they then transmit information to induce blood vessel cells and inflammatory cells to participate in VC (8). ECs are activated by endothelial progenitor cells (EPCs), oxidative stress, inflammation, autocrine and paracrine, mechanotransduction, hyperphosphatemia, and endothelial-to-mesenchymal transition (EndMT). These processes, which initiate or aggravate VC, are discussed in the review.

As one of the most important mechanisms for VC, EndMT has drawn significant interest. At embryonic stages, EndMT is involved in cardiovascular growth and differentiation; however, in adult stages, the same progress often induces different diseases such as pulmonary hypertension, myocardial fibrosis, AS, and VC (9). When ECs undergo EndMT, their cytoskeleton is disrupted, causing ECs to lose their well-structured appearance and turn into fibroblast-like cells. ECs acquire multidirectional differentiation potential, and their invasion and migration ability are increased (8). Notably, previous studies have reported that mesenchymal cells can be inversely transformed into ECs, thereby enhancing repair capabilities during acute cardiac injury (10). Therefore, reversing the process of EndMT is expected to become a new therapeutic strategy for VC, but the precise molecular mechanisms remain unclear. The application of lineage tracing and single-cell sequencing provides new approaches to EndMT research.

This article reviews VC pathogenesis and research progress, expounds the role of ECs in VC, and focuses on the EndMT mechanism and reversal, with a view to providing new ideas for VC prevention and treatment.

## Vascular calcification

### Definition and classification

**V**ascular calcification is an active extraosseous ossification progress regulated by multiple cell types and cytokines (11). It manifests excessive osteoblast synthesis and the mineralization of the calcified matrix (12). Abnormal mineral deposition gradually spreads throughout the blood vessels because of an imbalance between calcification inhibitors and promoters.

**V**ascular calcification classification provides researchers with different angles to study the mechanisms and effects of VC. Based on their volume and density, vascular calcified plaques are divided into microcalcification and macrocalcification (13). Microcalcification (<0.5 mm) and M1 macrophages are found in unstable plaques, whereas large calcified (>0.5 mm) plaques are accompanied by many M2 macrophages in stable plaques (13, 14). Microcalcification is primarily formed by degenerative inflammatory plaques, while large calcification formation is an active phenomenon similar to bone mineralization (13). In DM, the receptor for advanced glycation end (RAGE) and galectin-3 form calcified plaques of different sizes by regulating downstream signaling molecules, such as sortilin (15). Large-sized plaques with low calcium density are the most likely to induce acute cardiovascular events (16). In addition, osteocalcin is used as a biomarker of calcification stability to predict adverse events (17). As calcification is highly complex and genetic diversity in individual cells affects plaque structure, composition, and stability, the effects of calcification on plaque stability remain inconclusive.

The anatomical classification of VC is divided into intimal calcification, media calcification, valvular calcific aortic stenosis, and calciphylaxis (18, 19) (Table 1). Intimal calcification results from the combination of osteobiology and chronic inflammatory plaques (18, 20). FSS leads to intimal calcified plaque rupture because of the abnormal vascular wall resulting from fibrous atherosclerotic plaque and punctate calcifications (21). In the DM environment, macrophage galectin-3 is upregulated and promotes the migration of extracellular vesicles (EVs) derived from VSMCs to the intima, inducing vascular intimal calcification (22). Inflammation and lipid accumulation are not required for media calcification formation (18, 23). It was previously hypothesized that media calcification did not mediate thrombosis and vascular stenosis due to its special location (24). As a matter of fact, media calcification induces the loss of vascular elasticity and leads to heart failure, stagnation of blood, and thrombosis (24). Valvular calcific aortic stenosis is also an important category of the VC (25, 26). Currently, the only treatment for severe aortic valve calcification is aortic valve replacement. Calciphylaxis is a small artery calcification syndrome in end-stage kidney disease and is characterized by ischemic necrosis of the skin, subcutaneous adipose tissue, skeletal muscle, and other organs. As such, its prognosis is considerably poor; however, the exact pathogenesis remains unclear (26–29). Importantly, interdisciplinary clinical management can effectively improve the survival rate and life quality of patients with calciphylaxis (30).

**TABLE 1:** Classification of vascular calcification.

Classification	Location	Risk factors	Related diseases	References
**Intimal calcification**	Arterial intima	Cell senescence Oxidative stress Hyperlipidemic Apoptosis Inflammation	AS Metabolic syndrome T2DM	18–22

**Media calcification**	Arterial media	Cell senescence Oxidative stress Mechanical stress Elastase degradation	T2DM CKD Osteoporosis Marfan syndrome Pseudoxanthoma elasticum	18, 22–24

**Calcification of aortic valve**	Aortic valve	Cell senescence Hyperlipidemic Mechanical stress Inflammation	Hypertension Rheumatic heart Congenital bicuspid aortic valve	18, 25, 26

**Calciphylaxis**	Arterioles	Hyperphosphatemia Hypercalcemia Hypercoagulosis Hemodialysis	Renal failure Hyperparathyroidism Hypoparathyroidism VitaminKDeficiency Autoimmune disease Metastatic cancer	18, 26–29

**V**ascular calcification classification shows that VC morbidity and calcification patterns are distinct for different driving factors. The study of different VC models can identify specific mechanisms and therapeutic targets of different pathogenic factors.

### Overview of calcification mechanisms

**FIGURE 1:**
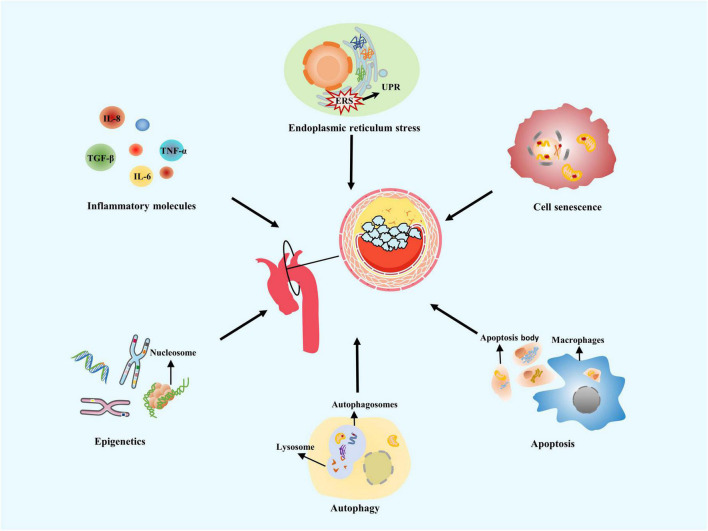
Mechanisms of vascular calcification. Vascular calcification (VC) often occurs at the aortic arch under rapid blood flow to generate strong shear forces on arterial walls. Common VC mechanisms include chronic inflammation, endoplasmic reticulum stress, cell senescence, autophagy, apoptosis, and genetic factors.

In recent years, breakthroughs in VC research have been mainly related to similarities between calcification and ossification mechanisms, and its active calcification regulation. Currently, the understanding of VC pathogenesis includes chronic inflammation, endoplasmic reticulum stress (ERS), cell senescence, autophagy, apoptosis, and genetic factors (26) (Figure 1). Different aspects of calcification mechanism interact with each other, perpetuate vascular damage, and eventually mediate VC (7, 31).

**V**ascular calcification is closely associated with inflammatory conditions. The most common intimal calcification mechanism occurs in inflammatory plaques, with lipid deposition as the core (32). Activated NOD-like receptor protein 3 (NLRP3) promotes the release of pro-inflammatory cytokines such as interleukin 1β (IL-1β), which induces the receptor activator of NF-κB ligand (RANKL) secretion and causes a series of inflammatory reactions to induce osteogenic differentiation in vascular cells (33). Plasma inflammatory markers, C-reactive protein (CRP), and IL-1β have been proved to be important predictors of VC (34). In view of harmful and irreversible effect of VC, predicting and preventing VC are also important for the treatment strategy.

Endoplasmic reticulum stress leads to cell death, when protein processing exceeds endoplasmic reticulum folding, and ubiquitin/proteasomal mechanisms fail to scavenge abnormal proteins (35). Stellate ganglion block has been reported to prevent ERS activation and improve VC, potentially presenting new ideas for VC treatment (36). Fibroblast growth factor 21 (FGF21) inhibits the upregulated ERS marker GRP78 in the calcified aorta, prevents apoptosis *via* the activation of CHOP and caspase-12 pathways, and improves VC in rats (37). Inflammation and ERS have been considered a defense response, but when they play an excessive role for an extended time, homeostatic balance becomes disrupted, and blood vessels are remodeled (38). ERS can interact with inflammatory cytokines and trigger pathways related to VC; this is one of the pathological basis of VC (39).

Cell senescence accelerates the progression of age-related diseases such as AS and DM, promoting calcification *via* inflammatory cascade reactions (40). Senescent cells demonstrate impaired DNA damage repair, loss of VSMC contractile phenotypes, and increased osteogenic differentiation (41). Autophagy degrades and recycles damaged cytoplasmic molecules and misfolded proteins *via* lysosomes (42). Specific reactive oxygen species (ROS) concentrations enhance the autophagy of arterial wall cells and reduce the release of matrix vesicles (MVs), thus inhibiting VC (43). Apoptosis is activated in AS plaques; the apoptosome, produced by apoptotic VSMCs, is similar to MVs, the nucleation sites for the formation of calcium crystals. When apoptotic cell phagocytosis becomes dysfunctional, necrosis may occur and potentially induce inflammation. Secondary necrosis of apoptotic VSMCs releases IL-1α and IL-β molecules (44).

A particular relationship exists between VC and genetic factors. Pseudoxanthoma elastosis is an inherited disease characterized by ABCC6 gene mutations. Progressive calcification of elastic fibers is observed in the skin, eyes, and vascular system (45). Generalized arterial calcification of infancy, an autosomal recessive disease, shows severe calcification of the aorta and coronary arteries (46). Phenotypic switching is often controlled by multiple VC loci, such as chromosome 9p21.3, 6p21.3, and 10q21.3 loci (47). Therefore, gene-susceptible phenotypes are often centrally expressed in a family. Patients with a family history of coronary heart disease have an increased likelihood of developing coronary calcification (48). Healthy femoral arteries with normal histological morphology exhibit an increased chance of developing VC in the absence of risk factors, which may be linked to the enrichment of genes related to bone development (49). Epigenetics consists of DNA methylation, histone modification, chromatin remodeling, and non-coding RNA. Epigenetics also affects calcification phenotypes without altering DNA sequences (50). Previous studies have demonstrated that disordered arterial blood flow regulates the promoter of mechanically sensitive gene hypermethylation to induce VC (51). An increase in histone 3 and 4 acetylation is one of the mechanisms of aortic valve calcification. C646, a histone acetyltransferase inhibitor, effectively reduces aortic valve calcification in mice (52).

The diversity of VC mechanisms provides several perspectives for the formulation of treatment strategies; nonetheless, no surgical methods are currently available to fully alleviate VC.

## Role of endothelial cells in vascular calcification

### Endothelial cells

#### Definitions and physiological functions of endothelial cell

The endothelium is a highly specialized and metabolically active interface between the blood and the vasculature (53). It has broad functions in regulating vascular tension, vascular integrity, and homeostasis. Notably, to cope with different stimuli, ECs display considerable plasticity, including osteogenic phenotype formation and EndMT processes (1).

Endothelium-derived NO acts on NO/CG/cGMP, the most important signaling pathway of blood vessels, to regulate vasodilation (54). Endothelium-derived hyperpolarization also include hydrogen sulfide (H_2_S), carbon monoxide, arachidonic acid metabolites, H_2_O_2_, and prostacyclin (PGI2). EC elicits endothelin 1 (ET-1), angiotensin II (Ang-II), and thromboxane A2 (TXA2), which regulate vasoconstriction and the balance of coagulation and fibrinolysis system (7, 55).

Vascular endothelium acts as a semipermeable barrier. Excessive endothelial permeability is caused by oxidative stress and inflammation (56). The loss of endothelial barrier integrity leads to vascular hyperpermeability and abnormal metabolism (57). ECs demonstrate strong repair capabilities (7). Static ECs can switch growth states, perform tissue regeneration or repair, and avoid the activation of coagulation cascades (7).

The vascular endothelium regulates the exchange of nutrients and metabolites. The major energy source for ECs is glycolysis, followed by fatty acid oxidation (FAO) (58, 59). ECs also have an active metabolism. Similarly, the raw materials, different products, and metabolic pathway regulation also affect EC proliferation and migration (7). PFKFB3 is the regulator of EC glycolysis. Pro-inflammatory cytokines decrease in mice with endothelial PFKFB3 gene knockout, slowing pulmonary hypertension progression to some extent (60).

#### Endothelial cell dysfunction

Endothelial cell dysfunction often contributes to disease development because of the diversity of endothelial functions. EC activation is not a binary process. When lesions accumulate, ECD occurs (53) and generates a series of phenotypes related to different pathological alterations. The processes are characterized by impaired endothelium-dependent vasodilation, increased endothelial permeability, dedifferentiation, and glycocalyx degradation (61). ECD is a critical risk factor for CVD (5).

Before VC imaging evidence and clinical symptoms appear, ECs have been dysfunctional. Thus, the timely detection of ECD is a major consideration for VC prevention (62). Moreover, ECD seems reversible after therapeutic intervention (63). For example, cholesterol-lowering and antioxidant therapies improve endothelium-dependent vasomotor functions and reduce CVD incidences (64). Currently, VC treatments focus on improving known risk factors, whereas specific endothelium-targeted therapy is rarely studied. Exploring new and stable ECD biomarkers can provide clinical guidance for the application of these endothelium-targeted drugs.

#### Endothelial cells subtypes

Recently, scientists reported that ECs in different organs and even in the same tissue were specific for different phenotypic and functional properties to meet different needs (65). EC subsets with significant cellular heterogeneity have been identified by single-cell sequencing of the aorta in C57/BL6 mice (66). The largest subgroup, EC1, specifically expresses VCAM-1, Cytl1, Clu, and Gkn3; EC2 expresses genes related to lipid transport (CD36, Fabp4, Lpl, and Gpihbp1) and angiogenic markers (Flt1), and EC3 expresses lymphoid identity markers (66). The endothelium on both sides of the aortic valve display different transcription profiles (1). However, it is unclear if the formation of these subtypes is the result of different EC origins or heterogeneous gene expression caused by the intravascular environment (66). Exploring different calcification mechanisms by tracking EC subsets can help reverse ECD.

### Contribution of endothelial cells to vascular calcification

**FIGURE 2:**
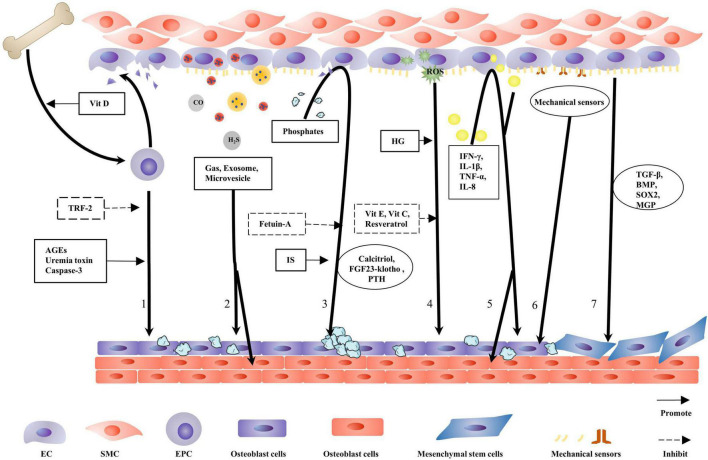
Contribution of endothelial cells to vascular calcification. When endothelial cells (ECs) are damaged, endothelial progenitor cells (EPCs) are released from the bone marrow into the circulation where EPCs repair the damaged blood vessel wall. This process is facilitated by physiological doses of vitamin D. TRF-2 delays EPC senescence and inhibits vascular calcification (VC). Caspase-3, advanced glycation end products (AGEs), and uremic toxins damage EPC function and transform the repair process into a calcification process. ECs secrete gas, exosome, and microvesicles, with gas directly regulating VC. Exosomes and matrix vesicles (MVs) carry molecules to regulate ECs and vascular smooth muscle cells (VSMCs) osteogenic differentiation. Hyperphosphatemia induce endothelial cell dysfunction and promotes VC; fetuin-A naturally inhibits calcification; and IS aggravates the harmful effects of hyperphosphatemia. The calcitriol–PTH–fibroblast growth factor 23 (FGF23)–klotho axis regulates the metabolism and calcification of calcium and phosphorus. HG is more conducive to reactive oxygen species (ROS) production, and antioxidants alleviate VC. Several inflammatory factors damage ECs to promote VC. Several mechanical receptors are present on the EC surface, detecting changes in blood flow shear stress and determining osteogenic differentiation in the vascular endothelium. ECs that have undergone endothelial-to-mesenchymal transition are disarranged, barrier function is diminished, migration and invasion capabilities are increased, and VC is more likely to occur.

The triad of mesenchymal cells, monocytes, and ECs control *in situ* and ectopic mineral metabolism (66). This review focuses on the role of ECs in VC. Osteocalcin expression in ECs is increased in the area where the highest mechanical stretching occurs, suggesting that ECs have the potential for osteogenic differentiation (67). In calcified arteries, co-expression of endothelial and osteogenic markers is observed by immunofluorescence staining (6). The main mechanisms allowing ECs to promote VC include EPC activation, oxidative stress, inflammation, autocrine and paracrine functions, mechanotransduction, hyperphosphatemia, and EndMT (Figure 2). Different mechanisms inducing endothelial dysfunction normally occur concurrently or act as concomitant triggers. ECs secrete gas signaling molecules and vasoactive substances to regulate inflammation, while inflammation also facilitates endothelial secretory function (68). Similar crosstalk also occurs between hyperphosphatemia and inflammation, mechanotransduction, and EndMT (69, 70). This indicates an intricate interaction network among those mechanisms and eventually leads to VC.

#### Endothelial progenitor cells

Endothelial progenitor cells from the bone marrow comprise an important source of healthy ECs. EPCs repair damaged blood vessels and have the potential to differentiate into osteoblasts (7, 71). VC is considered an acquired stem cell disorder. Pluripotent cells are susceptible to phenotypic changes induced by osteogenesis within the context of VC (6).

Physiological vitamin D concentrations alter RNA expression profiles in EPCs (72). Vitamin D promotes EPC migration from the bone marrow to blood vessels by affecting metalloproteinase (MMP) and guanosine triphosphate (GTP) activity, as well as increasing EPC adhesion to the injured endothelium (72). In DM, advanced glycation end products (AGEs)bind to their receptors in the membrane, stimulate EPC apoptosis *via* the mitogen-activated protein kinase (MAPK) pathway, reduce NO release, and promote the osteogenic differentiation of EPCs (73). In CKD, due to mineral metabolic disorders and uremic toxins, EPC counts decrease, while osteogenic phenotypic EPCs increase (74). Circulating osteogenic EPC counts may be used as VC markers (75). In patients with AS, EPC caspase-3 activity is increased, suggesting apoptosis initiation. TRF-2, which is related to telomere length and function, is downregulated in AS and induces senescence and apoptosis in EPCs (76).

Several similarities exist between VC and bone formation. VC formation partly depends on bone progenitor cells (6), the source of which are ECs. Matrix Gla protein (MGP) is vital for bone progenitor cell formation. MGP overexpression in *Ins2Akita*^/+^ mice limits pluripotent EPC production and VC. Moreover, the expression of pluripotent markers—SOX2, Nanog, and Oct3/4—in the aorta, accompanied by MGP deletion, is increased by 49% (6).

Distinct from reversal and regression after calcification formation, the beneficial effects of EPCs on VC seem to act in the initial disease development stages. Thus, treatment strategies targeting EPCs are required to avoid further damage.

#### Autocrine and paracrine

Under physiological conditions, ECs secrete different vasoactive substances to maintain vascular homeostasis (5). When the endothelial function is damaged, disordered secretory factors promote VC. Previously, ECs in the aorta of *SHR* rats secreted MMP2 and MMP9 to induce calcium deposition and osteogenic transformation of VSMCs (4).

Extracellular vesicles (EVs) mainly consist of exosomes and microvesicles, which carry proteins, lipids, or nucleic acids to target sites (77). In a high-glucose (HG) environment, ECs release exosomes containing the VCAN protein to induce VSMC mitochondrial dysfunction and promote VC (78). In addition, exosomes carry Notch3 and circRNA-0077930, which induce VSMC senescence (79, 80). Aging ECs produce microvesicles, which contain high levels of annexin, bone morphogenetic protein (BMP), and calcium to promote VSMC calcification (81). ECs cultured in HG release EVs, which contain highly activated NADPH oxidase, inducing EC activation and high ROS levels. This progress has also been confirmed in AS plaques in *ApoE*^–/–^ mice (82).

MicroRNAs have important roles in inflammation, metabolism, and CVD. Benefiting from microvesicle packaging, extracellular microRNA can survive in a highly active RNase environment. MicroRNAs carried by EC-derived cell-specific microvesicles are closely related to the AS inflammatory environment, particularly the let-7 microRNA family (83). EC-specific miR-126 has been shown to inhibit VCAM-1 expression, control vascular inflammation, and upregulate SPRED1 to promote endothelial repair and antagonize VC (84). EV research is highly topical; EVs become viable diagnostic VC biomarkers and important drug therapy targets. Similarly, EVs have the potential to become new drug carriers targeting different diseases.

In addition, ECs secrete gas signaling molecules (e.g., H_2_S, NO, and carbon monoxide (CO)) and vasoactive peptides to regulate VC development (26). Appropriate H_2_S concentration inhibits inflammation by reducing EC adhesion to leukocytes, scavenging free-radicals, and inhibiting NF-κB (68).

Endothelial cells synthesize and secrete some signaling molecules and various other chemicals to regulate VC formation. Therefore, investigating EC secretory functions could be significant for controlling VC.

#### High phosphate

Phosphate deposition is a sign of VC and is observed in all VC types. Excessive deposition of endogenous minerals phosphorus and calcium stimulates transformation to the osteogenic phenotype, damages endothelium-dependent vasodilation, and promotes AS and VC (85, 86).

Extracellular phosphorus contributes to the formation of calciprotein particles. Calciprotein particles exert strong cytotoxic effects, causing inflammation and inducing EC apoptosis. Fetuin-A resists these effects by binding to calcium phosphate (87). The uremic toxin indolyl sulfate (IS) relies on the JNK/Pit-1 pathway to enhance the effect of serum phosphorus on VC (88). Inorganic pyrophosphate is an important anti-mineralization factor in plasma. Excessive VC has been observed in a mouse model of Hutchinson–Gildford progeria syndrome, mainly due to increased TNAP activity and cell mitochondrial dysfunction, leading to pyrophosphate deficiency (89).

Phosphorus levels in humans are typically regulated by the calcitriol–parathyroid hormone (PTH)–fibroblast growth factor 23 (FGF23)–klotho axis (90). Overloaded phosphate increases the serum levels of FGF23, which regulates phosphate metabolism by influencing Npt-2 activity and inhibiting calcitriol synthesis (91). Compensatory increases in serum FGF23 and PTH in CKD occur much earlier than calcium and phosphate, which exemplifies the homeostatic protective effects of the body (91).

The direct effect of FGF23 on PTH has been found to require the dose-dependent downregulation of PTH mRNA expression (92). PTH stimulates osteocytes to secrete FGF23 by activating the protein kinase A and Wnt signaling pathways. Klotho is a highly expressed protein in the parathyroid gland and regulates FGF23 signal transduction (92, 93). Serum iron, erythropoietin, and insulin-like growth factor-1 are involved in phosphate regulation, and their regulatory mechanisms are closely related to FGH23 (90). These observations suggest that plasma FGF23 can function as an independent VC biomarker (94).

Klotho and FGF23 function together to maintain calcium and phosphate homeostasis. Klotho alleviates EC senescence by inhibiting nuclear factor-kappa B binding to nuclear DNA (95). It competitively binds FGFR1 (IIIc) to generate a high affinity between FGF23 and FGFR1 (IIIc). Klotho converts a co-receptor of FGF and FGFR1 (IIIc), into a specific receptor for FGF23 (90, 96). Heparin and glycosaminoglycan then effectively stabilize the FGF23–klotho–FGFR1 (IIIc) complex (96). Also, klotho increases urinary phosphate excretion and inhibits phosphate uptake by VSMCs (91).

The pathological deposition of minerals is an indispensable VC process. A number of diseases are closely associated with calcification, promoting the abnormal deposition of phosphate. Drug treatment for these diseases alleviates VC. Can these drugs be used to treat VC alone? This article proposes ideas for drug research and repurposing old drugs.

#### Oxidative stress

Oxidative stress destroys the redox dynamic balance by increasing ROS production and reducing ROS clearance, which are major factors implicated in EC damage (97). ROS molecules mainly include superoxide anion (O^2–^), hydrogen peroxide (H_2_O_2_), hydroxyl radical (OH^–^), and ozone (O_3_), which are characterized by high chemical reaction activity.

Hyperglycemia increases the concentration of ROS levels in ECs (98). In DM, hyperglycemia injures ECs *via* three biochemical pathways. Thenoyltrifluoroacetone (TTFA) is an inhibitor of complex II and significantly reduces ROS levels in HG environments. The pathway blocker 4-hydroxycyanocinnamic acid inhibits hyperglycemia-induced ROS production by weakening the TCA cycle, an important source of ROS. These molecules normalize mitochondrial ROS levels and block the biochemical pathway that potentially damages ECs (99).

Nitric oxide synthase (eNOS) maintains vascular homeostasis and protects EC. When cardiovascular risk factors interact, NADPH oxidase activity increases, producing large amounts of O^2–^ to manage environmental changes, while eNOS activity is upregulated. Oxidative stress uncouples eNOS, prompting its change from a NO-producing enzyme to an O^2^-producing positive feedback factor. Oxidative stress also destroys tetrahydrobiopterin, an eNOS co-factor. Such oxidative stress events also reduce NO bioavailability (100). Asymmetric dimethylarginine (ADMA) is an endogenous inhibitor of NO synthase and a risk factor for VC, while ROS enhances the activity of key enzymes producing ADMA (101).

Unequivocally, antioxidant stress endows VC with specific benefits. Several studies have demonstrated the protective effect of resveratrol on VC (102). Low doses exhibit antioxidant and anti-inflammatory properties, directly remove hydroxyl groups and superoxide, limit lipid peroxidation processes, upregulate eNOS activity, and confer protective properties on the endothelium (103). Other plant metabolites and vitamins, including quercetin, curcumin, and cordycepin, have similar functions (104). However, their specific clinical effects require further exploration.

#### Inflammation

Cardiovascular disease (CVD) mortality is significantly increased in patients with rheumatoid arthritis and may be related to a continuous increase in inflammatory factors, promoting VC (105). ECD is characterized by the upregulation of chemokines and adhesion molecules and the formation of a pro-inflammatory environment in blood vessels (106). These findings indicate that the inflammatory environment is closely related to vascular remodeling and VC.

Inflammation activates ECs and upregulates calcification-related genes (4). Inflammatory calcified plaques are significantly higher in DM, suggesting that the unbalanced environment during DM is more conducive to chronic inflammation processes. RAGE is significantly increased and mediates NF-κB activation to regulate corresponding genes (53, 107). RANKL promotes BMP release from ECs, while VSMC exposure to RANKL-treated EC-conditioned medium induces RUNX2 and ALP expression. NF-κB is an important pharmacological target (53). The IL-6 system is also positively correlated with ECD and AS, as well as accelerates the ROR2/WNT5A pathway in a STAT3-dependent manner to promote ectopic calcification (108).

Inflammatory cytokines not only directly promote EC osteogenic differentiation but also indirectly promote VSMC osteogenic differentiation *via* ECs. IL-8 acts as signals for EC-VSMC interaction in VC when induced by uremic toxins. ECs secrete IL-8 to downregulate OPN in VSMCs and consequently accelerate calcium deposition (88).

Any of the three major inflammatory factors—IFN-γ, IL-1β, and TNF-α—can injure the endothelium, reduce FGFR1 expression, and promote VC (109). Studies report that the dietary supplementation of omega-3 fatty acids produces resolvin E1, which mediates inflammation *via* ChemR23 receptors (110). Therefore, the benefits of polyunsaturated fatty acid toward VC deserve further study.

Controlling inflammation is vital, but controlling a single inflammatory factor clearly bears no practical significance for preventing and reversing VC (109). The general application of inflammatory inhibitors fails to adequately control vascular inflammation and VC because of the complexity of the latter.

#### Mechanotransduction

Vessel branches and corners are more likely to experience uneven and turbulent flow and are thus more prone to VC. Human blood flow is divided into unidirectional streamlined laminar flow (UF) and disturbed blood flow (DF). The former exerts antioxidant and anti-inflammatory effects, while the latter usually occurs in areas with branches and sharp turns, where it induces activation, amplifies EC responses to inflammation, and promotes calcification (111). Abnormal mechanical stress induces calcified plaque growth. Thrombi produced by plaque rupture spread to healthy tissue to form new diseased tissue, suggesting that calcified plaques are as difficult to control as cancer (14).

Arterial tissue responds to shear stress through structural changes and chemical reactions, which partly depend on mechanically sensitive cell ECs (14, 109). Different mechanical sensors exist on EC membranes, including primary cilia, glycocalyx, fossa, ion channel, integrin, PlexinD1, reticulin D1, and GPR68. The targeted regulation of these sensors improves endothelial function and reduces VC (111, 112). In ECs, piezo1 is an ion channel regulated by shear stress; its activation destroys the EC barrier, causing high vascular permeability and thus promotes VC (113).

The endothelial glycocalyx covers the entire endothelial surface and responds to the FSS. The endothelial phenotype is maintained by microstructures, including surface glycoprotein, proteoglycan core protein, sialic acid glycoprotein, and glycosaminoglycan (114). In Sprague–Dawley male rats fed a high-fat diet, the endothelial glycocalyx decreased significantly, and the endothelium was impaired, preventing vasomotor regulation *via* mechanotransduction and decreasing the release of endothelial protective factor NO (115). Different substances, such as heparin and MMP inhibitors, have been reported to help repair and regenerate the sugar calyx and inhibit VC progression (114).

VCAM-1 (EC1 marker) and CD36 (EC2 marker) were used to localize ECs in the mouse aorta; a clear boundary between high CD36 and high VCAM-1 was observed in the transition from the minor curvature to the large curvature of the aortic root; meanwhile, the EC morphology on both sides of the region varied and might have resulted from shear force-driven EC heterogeneity (66). With increased in-depth studies on single-cell sequencing and metabonomics, more regulatory factors are expected to be identified.

## Role of endothelial-to-mesenchymal transition invascular calcification

### Definition and characteristics of endothelial-to-mesenchymal transition

After a series of molecular events, ECs gradually lose EC lineage markers and acquire the characteristics of other cell lineages; this cellular reprograming is called EndMT (1, 109, 116, 117). The expression levels of newly formed endothelial markers VE-cadherin and CD31 decreased, whereas those of mesenchymal markers αSMA, N-cadherin, calmodulin, and FSP-1 increased. At the same time, the expression of several trigger factors, including Slug, Twist, and KLF4 in EndMT, can be monitored (1, 6, 118). Currently, no consensus has been reached on the definition of EndMT at the molecular level, impeding the establishment of a standardized model system and reducing the comparability and repeatability of experiments.

During embryonic development, EndMT occurs at the neural crest, heart valve, and neovascularization (119); in adults, the process is often associated with CVD characterized by vascular degeneration and fibrosis. During EndMT, EC polarity disappears; barrier functions decrease; migration and invasion increase; and proliferation and contraction are strengthened (1, 109). EndMT not only participates in normal tissue development; it also promotes disease progression and depends on timing and location (1, 119). Undoubtedly, EndMT mechanisms in the normal physiological state have particular relevance for pathological state mechanisms.

People have a clearer understanding of epithelial-to-mesenchymal transition (EMT), including reversible changes between EMT and mesenchymal-to-epithelial transition, than EndMT (116). As ECs comprise an epithelial cell subtype, applying well-known EMT mechanisms and targeted therapies to EndMT provides a good way to understand EndMT.

### Contribution of endothelial-to-mesenchymal transition to vascular calcification

**FIGURE 3:**
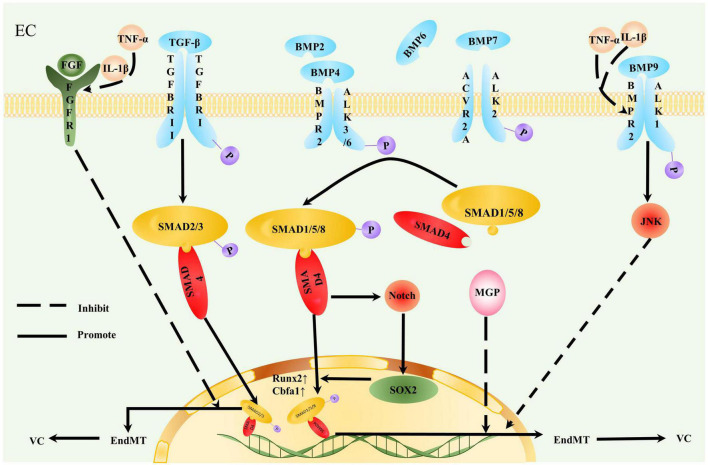
Contribution of endothelial-to-mesenchymal transition to vascular calcification. Endothelial cells (ECs) undergoing endothelial-to-mesenchymal transition (EndMT) are more likely to participate in vascular calcification (VC). TGF-β and bone morphogenetic protein (BMP) act on a tetrameric receptor complex to induce Smad-dependent signaling and regulate VC. TGF-β induces TGFBRII to activate TGFBRI. Type I receptors recruit and activate downstream Smad2/3/4 and Smad protein complexes to gather in the nucleus and act as transcriptional factors, thereby promoting VC. Inflammation stimulates decreased FGFR1 expression levels in EC. Interrupted fibroblast growth factor (FGF) signal transduction increases TGFBRI expression, loses the effective inhibition of TGF-β cascade reaction, and initiates EndMT. BMP transmits osteogenic signals by binding to activin receptor-like kinase (ALK)1/2/3/6 and phosphorylating Smad1/5/8. Smad1/5/8 and Smad4 form a quaternary complex isomer complex and translocate to the nucleus to promote osteogenic gene Runx2/Cbfa1 expression. The inflammatory factors, TNF-α and IL-1β, downregulate BMPR2 in aortic ECs, rendering ECs sensitive to osteogenic differentiation promoted by BMP9. TNF-α and IL-1β also promote EndMT by reducing JNK signal transduction. The calcification inhibitor matrix Gla protein (MGP) suppressed the effect of BMP2/4. Increased BMP4 expression also induces MGP expression; therefore, this negative feedback inhibits BMP2 and 4 osteogenesis. MGP deletion promotes EndMT. SOX2 is closely related to the osteogenic differentiation of ECs. BMP enhances SOX2 expression induced by Notch signaling, and SOX2 amplifies the osteogenic effect of the BMP4 signaling pathway.

Endothelial-to-mesenchymal transition generates mesenchymal cells that can potentially differentiate into bone lineage and cartilage lineage cells, which provide an important cell source of VC (1, 119, 120). Evidence suggests that cadherin-11 in interstitial tissue prompts mesenchymal cells to differentiate into osteoblasts and chondrocytes, thus promoting VC (121). The level of EndMT in ECs exposed to Y-channel disorder blood flow is significantly increased, and calcium deposition is detected (122). Thus, EndMT is induced and regulated by different cytokines, transcription factors, and microenvironment changes, thus mediating the inhibition of endothelial genes, providing osteoblasts, and promoting VC (Figure 3).

TGF-β and BMP are important factors inducing EndMT (119). BMP2/4/6/7/promotes EndMT and induces EC calcification (119). BMP transmits osteogenic signals by binding to activin receptor-like kinase (ALK)1/2/3/6 and phosphorylating Smad1/5/8. Smad1/5/8 and Smad4 form a quaternary complex and translocate to the nucleus to regulate osteogenic gene expression (14, 123). BMPI receptor ACVR1 mutation is the main reason for FOP ectopic calcification (124). In an investigation of animal model- and patient-derived tissues, the pro-inflammatory cytokines TNF-α and IL-1β downregulated BMPR2 in aortic ECs, rendering ECs sensitive to osteogenic differentiation promoted by BMP9; furthermore, EndMT was promoted by reducing JNK signaling (123). BMPR2 integrates TGF-β/BMP and EC inflammatory signals, exerting protective effects on VC. BMPR2 deletion increases bone mass in experimental mice. Therefore, drugs enhancing BMPR2 expression should be developed to limit EndMT (123).

Inflammation decreases FGFR1 expression levels in ECs. FGF signal interruption increases TGFβR1 expression and initiates TGF-β cascade reaction and EndMT (109, 125). ECs undergoing EndMT express high levels of leukocyte adhesion molecules and become strong pro-inflammatory cells (126). Inflammation and EndMT form a positive feedback loop that destroys vascular homeostasis. The inhibition of the TGF-β signaling pathway on the endothelium induces VC regression in mice (109). With the complexity and importance of TGF-β signaling considered, this pathway is worth exploring for the treatment of VC.

Several factors are important regulators of TGF-β and BMP signaling. MGP is a calcification inhibitor that suppresses BMP2, the main regulator of Runx2/Cbfa1 expression and an effective osteogenic inducing factor (127). BMP4 stimulates EndMT by activating ALK2 (6). Increased BMP4 expression also induces MGP expression, and this negative feedback inhibits BMP2/4 osteogenesis (127). When MGP is deleted, EndMT is generated, widespread BMP signaling is promoted, and VC rapidly develops (119). In MGP transgenic mice, MGP overexpression inhibits BMP activation by reducing ALK1 and vascular endothelial growth factor (6, 128). Notably, the formation of AS lesions is decreased in MGP-deficient mice may be due to a sharp reduction in the expression of endothelial adhesion molecules, resulting in decreased inflammatory cell infiltration in the arterial wall (6).

*In vitro* studies report that the expression of SOX2 and endothelial markers in embryonic stem cells is time-consistent. By contrast, when small interfering RNA (siRNA) molecules are used to deplete SOX2 transcripts in ECs, EC differentiation decreases, suggesting that SOX2 is closely related to this process and participates in EndMT (119, 129). BMP induces SOX2 expression by enhancing Notch signaling, and RBPJκ deletion inhibits SOX2 induction (129). SOX2 amplifies the osteogenic effect of the BMP4 signaling pathway (129). β-Blockers are suggested to eliminate SOX2 induction in the arterial endothelium and inhibit EndMT to further limit VC; this process is related to a significant decrease in RBPJκ expression and its subsequent binding to the SOX2 promoter (129).

The key role of the activation of specific serine proteases mediated by SOX2 in EndMT initiation has been proposed (130). Serine proteases degrade the internal elastic plate, which exposes VSMCs to harmful stimulation and increases the expression of two osteogenic EC markers, namely, Cbfa1 and osterix (130). Serine protease may regulate the conversion between endothelial and mesenchymal cells by regulating SOX2 (130, 131). Thus, the considered regulation of serine proteases can help prevent VC. Twist1 and SOX2 regulate each other and together promote EndMT. NOTCH signaling, hypoxia, and FGF also regulate the EndMT process in the TGF signaling pathway (132).

The WNT pathway also participates in mesenchymal transition, with the involvement of ligands Fz, LRP, GSK-3, and β-catenin in pathway activation. Once activated, twist transcription factors promote EndMT (14). These ligands can thus be targeted to inhibit the WNT pathway.

### Reversal of endothelial-to-mesenchymal transition

Gene expression patterns in EndMT cells differ from those in EC and mesenchymal cells, suggesting that EndMT is not a complete cellular transformation. Therefore, the existence of the intermediate state renders VC regression possible by reversing EndMT (94). Based on lineage-tracing studies, ECs express mesenchymal genes during embryonic development, which cannot be transformed into myofibroblasts; however, during adult cardiac fibrosis, mesenchymal genes are not even expressed (106). Therefore, the specific role of EndMT and the relationship between EC and mesenchymal cells require further investigation.

In a mouse model of heart failure, QishenYiqi inhibits inflammatory reactions, activates the NO-cGMP-PKG pathway, significantly reduces elevated EC adhesion molecules and EndMT markers, and critically reverses EndMT (133). BRD4 is the “reader” of lysine acetylation; the BRD4 inhibitor JQ1 downregulates EndMT-related transcription factors, attenuates EndMT, and induces EC migration by TGF-β (134). The DNA hydroxymethylase TET2 reversed EndMT-induced shear stress to a certain extent. Lentivirus transfection studies have shown that TET2 overexpression upregulates the EC markers, VE-cadherin, and CD31; downregulates the interstitial cell markers, vimentin, and α-SMA; and reduces atherosclerotic plaque synchronously (135). Icariin reverses ox-LDL-induced EndMT by regulating the expression of the H19/miR-148b-3p/ELF5 axis (136). Although EndMT research remains limited to the laboratory, the development of material science strategies (see below) transforms this research at the molecular and genetic levels.

## Role of endothelial cells in the treatment of vascular calcification

### Statins

Statins prevent CVD and reduce the incidence of coronary events in patients with DM; these therapeutic effects arise from improved endothelial function. Statins inhibit the synthesis of isoprene-like intermediates blocking the downstream Rho signaling pathway (137). The Rho family affects specific functions related to the shape, movement, secretion, and proliferation of ECs; thus, statins exert multiple effects on blood vessels (137).

Statins improve endothelium-dependent vasodilation functions that primarily depend on the upregulation of EC-based KLF2 levels to enhance eNOS activity (138), increase NOS, reduce oxidative stress, and inhibit the effect of endothelin (ET-1) (64, 137). Simvastatin significantly increases eNOS expression and activity and restores NO production (139).

Statins also inhibit the release of inflammatory cytokines and reduce inflammation stimulation toward ECs. However, frequent statin use in DM patients accelerates coronary artery calcification (137, 140). Low statin concentrations inhibit calcification, whereas harmful doses of simvastatin and lovastatin increase BMP2 expression and increase ectopic mineral deposition (141).

Briefly, statins exert certain protective effects on ECs; their overall benefits cannot be discounted. Therefore, considered doses should be selected as indicated, with a view to optimizing endothelium-targeting effects and improving VC outcomes.

### Hypoglycemic drugs

Diabetes is a risk factor for CVD. HG and insulin resistance environments generate persistent inflammation, oxidative stress, ECD, and mineral metabolism disorder; they also increase bone progenitor cell release into the circulation (98). Within this context, antidiabetic drugs could benefit ECD and VC.

Insulin is a widely used hypoglycemic drug. It stimulates the eNOS cofactor tetrahydrobiopterin, increases PI3K pathway activity, increases endothelium-derived NO, and improves endothelial function (142). However, insulin glargine has been reported to downregulate osteoprotegerin and promote VC *in vitro* (143). This finding is contrary to reports suggesting that hypoglycemic drug alleviates VC. Therefore, further research is needed.

Metformin promotes endothelial function as it improves hyperglycemia. The drug reduces osteogenic factor expression and inhibits VC induced by hyperphosphatemia *via* AMPK-RANKL signal transduction (102). Additionally, metformin increases eNOS activity and prevents VC *via* the AMPK-eNOS-NO pathway (144). It alleviates VSMC calcification induced by β-glycerophosphate, which may be related to autophagy *via* the AMPK/mTOR signaling pathway (145). Endothelial protection and VC alleviation, mediated by metformin, include several AMPK-mediated interactions. Therefore, AMPK can be a potentially viable therapeutic target.

In addition, several hypoglycemic drugs such as glucagon-like peptide 1 receptor agonists, sodium–glucose cotransporter 2 inhibitors, and dipeptidyl peptidase-4 inhibitors exhibit different endothelial and cardiovascular protective mechanisms (145). For instance, evogliptin significantly reduces calcium deposition and inhibits inflammatory cytokine in eNOS^–/–^ mice (146). Weight loss and diet regulation are also important hypoglycemic therapies as they reduce blood pressure damage, EC mechanical stress, and the spread of VC (14).

### Anti-inflammatory and antioxidant stresses

Inflammatory factors exert a damaging effect on ECs. With this finding considered, the development of major inflammatory factor (IL-6) inhibitors can alleviate VC (108). DMARDs reduce AS by inhibiting systemic inflammatory responses (108). Clinical trials have reported that Canakinumab significantly reduces IL-6 and CRP levels and inhibits inflammatory cells and EC adhesion; however, it increases the risk of infection and septicemia to a certain extent (147).

Aspirin, a non-steroidal anti-inflammatory drug (NSAID), improves improve endothelial function in AS and DM (148). However, in an AS multi-ethnic study of 6814 patients, baseline NSAID use exerts no significant protective effect on VC (149). Cyclooxygenase 2 (COX2) is an important target of anti-inflammatory drugs, but research suggests that the COX inhibitor celecoxib promotes aortic valve calcification (150). Contrary results also indicate that COX2 expression is increased in valvular calcification areas in klotho-deficient mice; in addition, feeding celecoxib to mice reduces calcification and blocks osteogenic gene expression (151).

Colchicine therapy inhibits endothelial inflammation *via* theNLRP3/CRP pathway. Studies have confirmed the beneficial effect of colchicine in AS (152). Soybean isoflavones generate antioxidant and anti-inflammatory effects and induce NO production, which contributes to the recovery of damaged endothelial function (153). Human umbilical vein ECs treated with genistein tend to accelerate autophagy and delay aging, which processes are related to the SIRT1/LKB1/AMPK pathway (154). Natural antioxidants exhibit considerable therapeutic effects, but the effectiveness of new antioxidants has yet to be evaluated in further clinical trials.

### Regulation of mineral metabolism

Some drugs can be used to regulate mineral metabolism. Calcium-based phosphate binders effectively control hyperphosphatemia, but their side effects induce hypercalcemia and promote VC, which should not be ignored (155).

The calcium-free phosphate binder Sveram significantly reduces different inflammation-related biomarkers (IL-6, IL-8, IL-10, CRP, TNF-α, and IFN-γ) and the mineral metabolism marker FGF-23; in addition, it reduces total cholesterol and increases HDL cholesterol. However, Sveram increases the endothelial markers implicated in cardiovascular events, including ICAM-1, VCAM-1, E-selectin, and L-selectin, leading to ECD (156, 157). Sveram has also been reported to increase flow-mediated vasodilation—that is, it enhances endothelial function (158). Some countries have approved the use of Sveram to delay VC progression in patients with CKD; however, Sveram does not significantly reduce mortality rates in dialysis patients (91).

Other phosphate binders such as lanthanum carbonate, nicotinamide, iron compounds, and bisphosphonates may clinically improve VC; however, their effectiveness and safety have yet to be confirmed (115).

### RNA interference technology

In personalized and accurate medical care, genome-based discoveries must be translated into clinical practice. ECs have powerful and diverse functions and complex pathogenic signals; thus, a desirable therapeutic outcome *via* the control of a single gene is difficult to achieve.

In mice, 7C1 nanoparticles efficiently transported RNAi molecules into ECs to simultaneously silence five endothelial genes (Tie1, Tie2, VEcad, VEGFR-2, and ICAM2). The procedure demonstrated high tolerance, selectivity, and efficiency (159). siRNAs were encapsulated into 7C1 nanoparticles and intravenously injected into HCHF-fed *ApoE*^–/–^mice. ECs absorbed the nanoparticles, which selectively inhibited TGF-β receptors. Finally, plaques and macrophages in diseased blood vessels were significantly decreased (160). Therefore, 7C1 nanoparticles can be used to study endothelial function and VC remission.

## Conclusion

In the modern era, VC incidence rates are expected to rise because of increased life expectancy. As a complex circulatory disease, calcification in varying volumes and densities affects atherosclerotic plaque stability, and its four anatomical types are closely related to different diseases. Early identification and treatment of individuals at risk for VC offers the potential to significantly reduce the mortality. The mechanisms of VC are complex and varied. Inflammation, ERS, cell senescence, autophagy, apoptosis, and genetic factors promote vascular remodeling and calcification. Although VC is a widely researched subject, the lack of strategies for its treatment suggests the need to further investigate its mechanism. The emergence of new technologies and the establishment of new *in vivo* and *in vitro* calcification models have laid the foundations for increased VC research.

In recent years, relationships between ECs and VC have gained extended research traction. The main mechanisms of EC promoting VC include EPC activation, oxidative stress, inflammation, autocrine–paracrine functions, mechanotransduction, hyperphosphatemia, and EndMT. Excessive imbalance of the internal environment leads to ECD and is involved in VC. The most effective approach to treating a disease is to stop its progression before it damages the body. Therefore, the exploration of VC-associated endothelial markers and the development of new targeted therapies for colorectal cancer are key research directions for overcoming VC.

The endothelium also exhibits plasticity. In EndMT, the protective and regulatory functions of ECs are substantially reduced, and ECs tend to perform osteogenic differentiation, an important pathological process of VC. EndMT is the focus of current studies; thus, its reversal is an attractive proposition for VC treatment.

Key to future VC treatment strategies is the comprehensive understanding of environmental factors and transcriptional and signaling mechanisms that drive phenotypic changes. The combined application of medicine and materials science is expected to bring hope for VC treatment.

## Author contributions

HJ conceived the topic and wrote the manuscript. LL and LZ provided help and advice on the manuscript, tables, and pictures. All authors contributed to editorial changes in the manuscript, read and approved the final manuscript.

## Conflict of Interest

The authors declare that the research was conducted in the absence of any commercial or financial relationships that could be construed as a potential conflict of interest.

## Publisher’s Note

All claims expressed in this article are solely those of the authors and do not necessarily represent those of their affiliated organizations, or those of the publisher, the editors and the reviewers. Any product that may be evaluated in this article, or claim that may be made by its manufacturer, is not guaranteed or endorsed by the publisher.

## Abbreviations

AGE: advanced glycation end products
ALK: activin receptor-like kinase
AS: atherosclerotic
BMP: bone morphogenetic protein
CKD: chronic kidney disease
CVD: cardiovascular disease
DM: diabetes mellitus
EC: endothelial cell
ECD: endothelial cell dysfunction
EndMT: endothelial-to-mesenchymal transition
eNOS: nitric oxide synthase
EPC: endothelial progenitor cells
ERS: endoplasmic reticulum stress
ET-1: endothelin-1
EV: extracellular vesicles
FGF23: fibroblast growth factor 23
FSS: flow shear stress
H_2_S: hydrogen sulfide
MGP: matrix Gla protein
NLPR3: nucleotide-binding oligomerization domain, leucine-rich repeat and pyrin domain-containing 3
NO: nitric oxide
PGI2: prostacyclin
RAGE: receptor for advanced glycation end
RANKL: receptor activator of NF-κB ligand
ROS: reactive oxygen species
VC: vascular calcification
VSMC: vascular smooth muscle cell.
